# Guidelines of the Polish Respiratory Society on the Diagnosis and Treatment of Progressive Fibrosing Interstitial Lung Diseases Other than Idiopathic Pulmonary Fibrosis

**DOI:** 10.3390/arm90050052

**Published:** 2022-10-04

**Authors:** Wojciech J. Piotrowski, Magdalena M. Martusewicz-Boros, Adam J. Białas, Adam Barczyk, Bogdan Batko, Katarzyna Błasińska, Piotr W. Boros, Katarzyna Górska, Piotr Grzanka, Ewa Jassem, Dariusz Jastrzębski, Janina Kaczyńska, Otylia Kowal-Bielecka, Eugeniusz Kucharz, Jan Kuś, Barbara Kuźnar-Kamińska, Brygida Kwiatkowska, Renata Langfort, Katarzyna Lewandowska, Barbara Mackiewicz, Sebastian Majewski, Joanna Makowska, Joanna Miłkowska-Dymanowska, Elżbieta Puścińska, Alicja Siemińska, Małgorzata Sobiecka, Renata Anna Soroka-Dąda, Małgorzata Szołkowska, Elżbieta Wiatr, Dariusz Ziora, Paweł Śliwiński

**Affiliations:** 1 Department of Pneumology, Medical University of Lodz, 90-153 Lodz, Poland; 2 3rd Lung Diseases and Oncology Department, National Tuberculosis and Lung Diseases Research Institute in Warsaw, 01-138 Warsaw, Poland; 3 Department of Pathobiology of Respiratory Diseases, Medical University of Lodz, 90-153 Lodz, Poland; 4 Department of Pneumonology, School of Medicine in Katowice, Medical University of Silesia, 40-635 Katowice, Poland; 5 Department of Rheumatology and Immunology, Faculty of Medicine and Health Sciences, Andrzej Frycz Modrzewski University, 30-705 Krakow, Poland; 6 Department of Radiology, National Tuberculosis and Lung Diseases Research Institute in Warsaw, 01-138 Warsaw, Poland; 7 Lung Pathophysiology Department, National Tuberculosis and Lung Diseases Research Institute in Warsaw, 01-138 Warsaw, Poland; 8 Department of Internal Medicine, Pulmonary Diseases and Allergy, Medical University of Warsaw, 02-091 Warsaw, Poland; 9 Department of Radiology, Voivodeship Hospital in Opole, 45-061 Opole, Poland; 10 Department of Allergology and Pneumonology, Medical University of Gdansk, 80-214 Gdańsk, Poland; 11 Department of Lung Diseases and Tuberculosis, Medical University of Silesia, 41-803 Zabrze, Poland; 12 Patient, not affiliated, 07-420 Kadzidlo, Poland; 13 Department of Rheumatology and Internal Medicine, Medical University of Białystok, 15-276 Białystok, Poland; 14 Department of Internal Medicine, Rheumatology and Clinical Immunology, Medical University of Silesia, 40-635 Katowice, Poland; 15 1st Lung Diseases Department, National Tuberculosis and Lung Diseases Research Institute in Warsaw, 01-138 Warsaw, Poland; 16 Department of Pulmonology, Allergology and Respiratory Oncology, University of Medical Sciences in Poznan, 61-701 Poznan, Poland; 17 Department of Rheumatology, Eleonora Reicher Rheumatology Institute, 02-637 Warszawa, Poland; 18 Department of Pathology, National Tuberculosis and Lung Diseases Research Institute in Warsaw, 01-138 Warszawa, Poland; 19 Department of Pneumonology, Oncology and Allergology, Medical University, Lublin, 20-090 Lublin, Poland; 20 Department of Rheumatology, Medical University of Lodz, 92-213 Lodz, Poland; 21 2nd Department of Respiratory Medicine, National Tuberculosis and Lung Diseases Research Institute in Warsaw, 01-138 Warsaw, Poland; 22 Department of Allergology, Medical University of Gdańsk, 80-214 Gdansk, Poland; 23 Patient, not affiliated, 02-673 Warsaw, Poland

**Keywords:** progressive fibrosing interstitial lung disease, progressive pulmonary fibrosis, GRADE, diagnosis, treatment, autoimmune diseases, PF-ILD, PPF

## Abstract

**Highlights:**

The working group of the Polish Respiratory Society (PTChP) developed guidelines for diagnosis and treatment of PF-ILD.

**What are the main findings?**

- A multidisciplinary team should be involved in the diagnosis and treatment of progressive pulmonary fibrosis.
- Nintedanib alone or in combination with immunomodulatory drugs is recommended for the treatment of PF-ILD, especially when an earlier solely immunomodulatory treatment was ineffective.

**What are the implications of the main finding?**

- This document is a guide for Polish medical personnel involved in the diagnosis and treatment of PF-ILD.
- The guidelines will serve as an aid for healthcare organizers in Poland on how to optimize the diagnostic and therapeutic processes for ILD and improve the access of patients to modern therapy.

**Abstract:**

The recommendations were developed as answers to previously formulated questions concerning everyday diagnostic and therapeutic challenges. They were developed based on a review of the current literature using the GRADE methodology. The experts suggest that PF-ILD be diagnosed based on a combination of different criteria, such as the aggravation of symptoms, progression of radiological lesions, and worsening of lung function test parameters. The experts recommend a precise diagnosis of an underlying disease, with serological testing for an autoimmune disease always being included. The final diagnosis should be worked out by a multidisciplinary team (MDT). Patients with an interstitial lung disease other than IPF who do not meet the criteria for the progressive fibrosis phenotype should be monitored for progression, and those with systemic autoimmune diseases should be regularly monitored for signs of interstitial lung disease. In managing patients with interstitial lung disease associated with autoimmune diseases, an opinion of an MDT should be considered. Nintedanib rather than pirfenidon should be introduced in the event of the ineffectiveness of the therapy recommended for the treatment of the underlying disease, but in some instances, it is possible to start antifibrotic treatment without earlier immunomodulatory therapy. It is also admissible to use immunomodulatory and antifibrotic drugs simultaneously. No recommendations were made for or against termination of anti-fibrotic therapy in the case of noted progression during treatment of a PF-ILD other than IPF. The experts recommend that the same principles of non-pharmacological and palliative treatment and eligibility for lung transplantation should be applied to patients with an interstitial lung disease other than IPF with progressive fibrosis as in patients with IPF.

## 1. Introduction

Interstitial lung diseases (ILDs) form a heterogeneous group of diseases of various etiopathogeneses, clinical courses, and prognoses [1]. 

The natural course of an ILD may have multiple scenarios. In some situations, lung lesions may regress, either spontaneously or as a result of treatment. Unfortunately, some cases present with lesions of an adverse, progressive nature, leading to advanced pulmonary fibrosis and, consequently, respiratory failure and death [2,3]. 

Progressive fibrosing interstitial lung diseases (PF-ILDs) form a diverse group of diseases, including entities with varying etiologies and clinical, pathomorphological, and radiological presentations [1,4]. An archetypal example of such a disease is idiopathic pulmonary fibrosis (IPF). IPF patients have pulmonary fibrosis with the pattern of usual interstitial pneumonia (UIP), and progression is included in the natural history of the disease [5,6,7]. This disease is limited to the lungs only, and its diagnosis requires the exclusion of other causes of fibrosis [5,6,7]. Current observations, which have been supported by clinical trials, show that IPF is characterized by unpredictable but progressive and significantly higher rates of decline in lung function compared to the general population, resulting in a median survival that is even reduced to 3–5 years [8,9]. A feature of progression can occur in diseases with a wide variety of etiologies, most commonly including fibrotic hypersensitivity pneumonitis (fHP), non-specific interstitial pneumonia (NSIP), systemic-autoimmune-disease-associated interstitial lung disease, unclassifiable interstitial lung disease (uILD), silicosis, and sarcoidosis [10,11,12,13,14]. Data from different reference centers indicate that progression in patients with interstitial fibrosis occurs in 31–52% of patients [10,11,12,13]. A multicenter survey conducted among physicians involved in diagnostics and treatment of ILDs indicated that 18–32% of patients with interstitial fibrosis other than IPF developed the progressive fibrosis phenotype [14,15]. 

Progression is associated with a poor prognosis, which is similar to the one observed in patients with IPF [3]. Anti-fibrotic agents used in this diverse group of patients have been shown to slow fibrosis progression to a similar extent to that seen in IPF patients [16,17]. The beneficial effect of anti-fibrotic drugs does not appear to be dependent on the underlying type of interstitial lung disease or radiological pattern of interstitial fibrosis (UIP vs. non-UIP) [16,18].

A major problem is that, at present, there is no generally accepted standard of care (both diagnostic and therapeutic) in patients with features of PF-ILD. At the moment of diagnosis of potentially fibrosing ILD, particularly at an early stage and with a short follow-up, it is difficult to assess and predict its further course. The PF-ILD rate is estimated based on retrospective analyses of patient groups with interstitial lung lesions who presented with such an unfavorable course of the disease. The results differ, which is not surprising, given the different selection criteria, as well as the different criteria for fibrosis progression [19,20,21,22]. 

Progression in the course of an ILD is not a marginal phenomenon because it is likely to affect every fifth or even every third patient with potentially fibrosing lung disease [14]. A significant percentage—as high as between 25 and 50% of patients with PF-ILD—did not receive any pharmacological treatment, and the time from the onset of the first symptoms to death was only 61–80 months [14]. Various reasons for not undertaking therapeutic intervention were determined, including disease advancement, intolerance toward medications, and lack of effective therapy in the analyzed period [14]. The poor prognosis of PF-ILDs other than IPF (which, in fact, is identical to the prognosis in IPF patients) and data from clinical trials demonstrating the beneficial effect of anti-fibrotic therapy (at this point, regarding nintedanib) support the isolation of this phenotype, which is common for the above-defined disease group, as a separate clinical entity requiring standardized and dedicated diagnostics and treatment [3,16,23]. 

The possibility of effective anti-fibrotic treatment is an opportunity for patients with progressive interstitial pulmonary fibrosis other than IPF to slow down the progression rate and possibly even prolong life. Therefore, standardization of the definition together with the identification criteria for PF-ILD is a key aspect of optimizing management in this patient group. 

A preliminary attempt to define PF-ILD by determining diagnostic criteria was performed at the stage of clinical trials that evaluated the effect of anti-fibrotic treatment [16,17]. Based on this experience, a proposal was formulated to define the phenotype within a specific ILD with features common to all ILDs that may develop progressive pulmonary fibrosis despite the use of conventional treatment [4]. 

To identify patients with the potentially poorest prognosis, the diagnostic criteria for PF-ILDs should include a combination of worsening radiographic lesions (CT), worsening lung function, and an increase in the severity of experienced symptoms [4]. As in other cases, the role of a multidisciplinary team, both at the stage of determining an appropriate, precise diagnosis of ILD and the stage of identification of the PF phenotype (assessment of disease progression or lack of efficacy of first-line treatment, dedicated to patients with a specific diagnosis), is emphasized. Previous observations indicate that PF-ILD may affect a significant proportion of ILD patients, and the need to identify and separate this group of patients is mainly based on practical evidence that anti-fibrotic treatment is effective in patients with progressive interstitial pulmonary fibrosis other than IPF. Standardization of the diagnostic criteria for PF-ILD would allow for an optimal identification and qualification for anti-fibrotic treatment of an adequate group of patients.

## 2. Materials and Methods

Polish recommendations for diagnostic and therapeutic management were developed by a team of Polish experts in the field of ILDs and systemic diseases. The initiative for the development of national guidelines was launched by a group of experts in interstitial diseases who participated in cyclical training for lung specialists due to the rise of unequal opinions on the diagnosis of progressive fibrosis in interstitial lung diseases other than IPF, eligibility for treatment, treatment regimens with anti-fibrotic agents, supportive and palliative treatment, and methods for monitoring the course of the disease. The initiative was supported by the Main Board of the Polish Respiratory Society. 

These guidelines were developed based on a worldwide literature review, taking into account the local circumstances resulting from the disparities in the Polish healthcare system, the lack of an official treatment program for patients with progressive fibrosis in the course of interstitial lung diseases other than IPF, and current problems with treatment reimbursement in Poland. 

Objectives of the guidelines:

1. To improve the quality and reliability of the diagnosis of progressive fibrosis in the course of interstitial lung diseases other than IPF;
2. To increase access to anti-fibrotic therapy by promoting diagnostic and eligibility criteria for treatment in Poland;
3. To identify health needs and deficiencies in the care of patients with progressive fibrosis in the course of interstitial lung diseases other than IPF.

These guidelines do not refer to the diagnostics and treatment of dedicated underlying diseases that reveal the PF-ILD phenotype, i.e., hypersensitivity pneumonia, systemic autoimmune disease, or sarcoidosis. In this respect, we refer to the pertinent guidelines and recommendations of the relevant scientific societies.

The group of patients covered by the guidelines includes patients with suspected or diagnosed progressive fibrosis in the course of interstitial lung diseases other than IPF. The target groups for which the guidelines were developed are physicians dealing with interstitial lung diseases—in particular, respiratory medicine specialists, rheumatologists, radiologists, pathologists, thoracic surgeons, respiratory rehabilitation specialists, healthcare organization specialists, and representatives of the National Health Fund and other institutions shaping health policy in Poland.

Members of the working team: The working team consisted of medical specialists in different fields and representing different expert centers, as well as patients. A literature review for individual problems was prepared independently by the authors designated to develop a specific assignment and independently by the author designated to perform the function of a librarian (A.J.B.). The authors W.J.P. and A.J.B. supervised the methodological consistency of the guidelines.

The team’s work was coordinated by the authors W.J.P. and M.M.M.-B.

The methodology was developed based on the “Methodical framework for developing diagnostic and therapeutic recommendations” issued by the Agency for Health Technology Assessment and Tariff System [24]. The guidelines were developed using the Grading of Recommendations Assessment, Development, and Evaluation (GRADE) methodology in a form similar to that used in the opinion made by the American Thoracic Society [25]. Issues were resolved with the Delphi method. At each stage of the development of the guidelines, the evaluation criteria described in the “AGREE II guideline assessment tool” [26] were also taken into account.

The main methodical problem, which is particularly significant in the context of the assessment of the quality of evidence, is the classification of the analyzed group of diseases. Namely, progressive fibrosis in interstitial lung diseases other than IPF is defined through the exclusion of a group of diseases with a shared phenotype of progressive pulmonary fibrosis that does not meet the diagnostic criteria for IPF, a that is disease strictly defined in line with current guidelines [7]. This division results in a large number of disease entities representing this group of diseases, as well as a considerable heterogeneity, resulting in methodological imperfections and consequent difficulties in their synthesis. 

The recommendations were divided into two parts: diagnostics and treatment. The experts prepared clinical problem proposals in the form of a list of questions. Proposed questions were sent to all members of the Editorial Committee, and the proposed clinical questions were assessed in terms of their significance after introducing preliminary modifications and corrections. The score proposed by the authors of the consensus by the Swiss Society of Lung Diseases [27] was used. The significance of individual questions was assessed according to a 9-point scale and was divided into the following categories: very significant questions (8–9 points), significant questions (6–7 points), and non-significant questions (less than 6 points). Only questions considered to be very significant or significant were the subject of further procedures.

Next, a working team was appointed to develop specific guidelines: introduction—M.M.M.-B., W.J.P., and A.J.B.; methodology—A.J.B. and W.J.P.; diagnostics—M.M.M.-B., K.G., J.M., J.M.-D., and B.K.; treatment—S.M., K.L., M.S., J.M., J.M.-D., and B.K.

A review of the available literature was conducted based on the Medline and Cochrane databases. The literature search was terminated on 31 December 2021. This is why we do not refer to the newest ATS/ERS/JRS/ALAT international guidelines on progressive pulmonary fibrosis in this document [28]. We also decided to stay with the term “progressive fibrosing ILD, PF-ILD” that is currently used worldwide, instead of changing it to PPF, as proposed by the authors of the most recent international guidelines. The quality of evidence was assessed as high, moderate, low, and very low. The strength of recommendations was assessed as strong or conditional [7]. The authors of each section developed the first version of the answer to the question asked and a brief introduction to its content. Subsequent versions were developed following discussions by electronic means and during face-to-face meetings. The initial version was sent to all authors of the guidelines for internal review. 

The final version was sent for external review. After final corrections, the work was sent for publication. The English-language version was adopted as the original version. 

The recommendations will be subject to scheduled review every five years or earlier if new evidence that has a significant impact on the state of knowledge concerning the diagnostics or treatment of progressive fibrosis in interstitial lung diseases other than IPF becomes available and the expert panel concludes that it considerably changes the meaning of the already issued guidelines and, therefore, requires an upgrade earlier than scheduled.

## 3. Results

### 3.1. Diagnostic Module

Question 1: **Can a combination of data indicating the increased severity of clinical symptoms, worsening of respiratory function parameters, and progression of lesions in high-resolution computed tomography (HRCT) serve as a basis for the diagnosis of progressive fibrosing interstitial lung disease (PF-ILD)?**

#### 3.1.1. Background

Interstitial lung diseases with progressive fibrosis are characterized by worsening lung function and progression of lesions visible on high-resolution computed tomography (HRCT), worsening of symptoms, deterioration of quality of life, and increased risk of death [1,2,29,30]. The INBUILD study, which evaluated the efficacy of nintedanib in the treatment of progressive fibrosing interstitial lung disease (PF-ILD), required a precise definition of progression, which is common for many different diseases [16,31]. The INBUILD study enrolled patients with any interstitial lung disease other than IPF that showed progression of lung disease despite the standard of care [16]. The eligibility criteria for the INBUILD study were the demonstration of the following in the 24 months before inclusion in the study: relative FVC loss by at least 10%; relative FVC decline by 5–10% with concomitant worsening of symptoms or increased severity of interstitial fibrosis lesions in HRCT; worsening of respiratory symptoms and increased fibrosis in HRCT. Given the positive results of the INBUILD study, the eligibility criteria adopted in this study became the basis for the proposed diagnostic criteria for PF-ILD [4]. A reduction in the pulmonary transfer rate for carbon monoxide (T_L,CO_) was not used among the eligibility criteria in the INBUILD study [16]. T_L,CO_ is a variable indicator with inter- and intra-laboratory variability [32], and an isolated reduction in T_L,CO_ may occur in vascular disorders or in patients with emphysema. However, in a specific clinical context, particularly in the case of the accompanying FVC loss, a decrease in T_L,CO_ may be considered an indicator of ILD progression [33]. 

Taking into account previously published proposal [4] PF-ILD should be considered when, over 24 months of observation the criteria of progression are fulfilled. However, due to practical reasons and feasibility of calculation we suggest using absolute decreases of FVC and T_L,CO,_ as described below:

1. Absolute decrease in FVC of ≥10% predicted;
2. Absolute decrease of 5–10% in FVC predicted and one of the following: ≥15% absolute decrease in T_L,CO_ predicted or intensification of symptoms, or increase in the extent of radiological changes in HRCT;
3. Intensification of symptoms and increase in the extent of radiological changes in HRCT.

Expert panel recommendations have been depicted in Box 1.

Box 1 **Diagnostic Module Recommendation 1.**

**Recommendation 1:**

**We suggest that the basis for the diagnosis of progressive fibrosing interstitial lung disease (PF-ILD) should be an increased severity of clinical symptoms, worsening of pulmonary function parameters, and progression of lesions in high-resolution computed tomography of the chest**.

*Evidence quality: very low*

**Strength of recommendation: conditional**

(voting results: strongly for—14 votes, conditionally for—15 votes, abstain from voting—0 votes, conditionally against—0 votes, strongly against—0 votes)

**Commentary:**  *All available clinical assessment tools should be used for progression assessment. While lung function parameters (e.g., FVC and T_L,CO_) constitute objective parameters, assessment of the severity of symptoms and radiological progression contain a large dose of subjectivism. Therefore, we suggest that these parameters should be treated inseparably.*

Question 2: **In each patient with fibrotic interstitial lung disease, should we aim at establishing a precise diagnosis of the underlying disease?**

#### 3.1.2. Background

ILDs form a heterogeneous group of over 200 diseases, most of which are rare [1]. Pulmonary fibrosis may result from the progression of many diseases with various etiopathogeneses, which are characterized—especially in the early period—by different clinical, radiological, or pathomorphological presentations. Since the features and consequences of advanced fibrosis appear to be common, regardless of the initial diagnosis of the underlying disease, the progressive fibrosis phenotype across individual diseases has been identified [4]. The separation of this common feature made it possible to change the concept of treatment of selected interstitial diseases that do not respond to conventional causal treatment (anti-inflammatory or immunosuppressive) and show a favorable response to anti-fibrotic treatment [16]. Many diseases, such as those representing autoimmune diseases, especially in the early development of lung lesions, should be treated with an anti-inflammatory or immunomodulatory therapy due to the potential reversibility of these lesions, as well as a beneficial effect on other extrapulmonary manifestations of the underlying disease. In hypersensitivity pneumonitis (HP), the determination of the nature and source of the antigen allows for its elimination, which may have a positive impact on the long-term prognosis [34]. Therefore, a precise diagnosis allows for optimal management in a given patient [4,35]. This principle also applies to the diagnosis of IPF, where anti-fibrotic treatment is the recommended treatment of choice, whereas anti-inflammatory and immunosuppressive therapies are associated with a significant risk of deterioration and should not be used [5,16]. Precise differential diagnostics completed by establishing a proper diagnosis and the implementation of optimal management at the early stage of the disease have a beneficial effect on slowing the rate of decline of lung function and improving prognosis [36,37,38,39]. Expert panel recommendations have been depicted in Box 2.

Box 2 **Diagnostic Module Recommendation 2.**

**Recommendation 2:**

**We recommend that in every patient with fibrotic interstitial lung disease, we should aim to establish a precise diagnosis of the underlying disease**.

*Evidence quality: very low*

**Strength of recommendation: strong**

(voting results: strongly for—19 votes, conditionally for—6 votes, abstain from voting—2 votes, conditionally against—2 votes, strongly against—0 votes)

**Commentary:**  *A precise diagnosis of interstitial lung disease is not always possible, even with the application of all available assessment methods. However, where possible, a precise diagnosis allows for optimal treatment and elimination of harmful environmental factors, which will potentially have a positive impact on long-term prognosis.*

Question 3. **Is it necessary to perform serological tests for autoimmune diseases in every patient with fibrotic interstitial lung disease of unknown etiology?**

#### 3.1.3. Background

Depending on the source, autoimmune diseases cause between 7.5% and 34.8% of interstitial lung diseases [40]. Lung involvement may be the first clinical manifestation of a systemic autoimmune disease [41]. Although many previous reports used the term “connective tissue disease-related ILD (CTD-ILD), taking into account wider spectrum of diseases, including rheumatoid arthritis, we decided to use in this document the term “autoimmune-related ILD (AI-ILD). 

International guidelines for IPF diagnosis [5], as well as the Polish Respiratory Society guidelines [7], recommend that serological testing should be performed in every case of an ILD of unknown etiology. In the case of the international guidelines, this recommendation was considered to be a motherhood statement [5]. The minimum extent of these tests is considered to be the assessment of the general titer of antinuclear antibodies (ANAs), rheumatoid factor (RF), and anti-cyclic citrullinated peptides (anti-CCPs) [5,7]. Antinuclear antibodies can be detected in up to 1/5–1/3 of IPF patients and approximately 1/5 of the healthy population [42]. Therefore, it should be emphasized that high titers of ANA antibodies at screening, especially without the detection of antibodies specific to individual diseases and without other clinical manifestations suggestive of a given disease, cannot serve as a basis for the diagnosis of systemic autoimmune disease. Although experts suggest [42] pre-determining the ANA titer with an indirect immunofluorescence screening assay and, only if positive, expanding diagnostics for the presence of specific antibodies directed against extractable nuclear antigens (ENAs), it should be remembered that in the case of some autoantibodies, such as Ro52 and antisynthetise antibodies (e.g., Jo-1), ANA screening may be negative. In addition, the absence of autoantibodies does not exclude the diagnosis of systemic disease, as it may be a seronegative case or antibodies may appear later in the development of a systemic autoimmune disease [42].

The diagnosis of a systemic autoimmune disease is based, apart from serological evaluation, on clinical presentation, imaging findings, and results of other investigations, such as capillaroscopy, while serological tests are some of many classification criteria for these diseases [43]. The participation of rheumatologists in the interpretation of serological test results and other components of diagnosis may be of key importance [44]. Expert panel recommendations have been depicted in Box 3.

Box 3 **Diagnostic Module Recommendation 3.**

**Recommendation 3:**

**We recommend that every patient with fibrotic interstitial lung disease of unknown etiology should undergo serological tests for autoimmune diseases**.

*Evidence quality: very low*

**Strength of recommendation: strong**

(voting results: strongly for—23 votes, conditionally for—5 votes, abstain from voting—1 vote, conditionally against—0 votes, strongly against—0 votes)

**Commentary:**  *Around 25% of PF-ILDs have an autoimmune cause. We have observed similar clinical and radiological pictures of many interstitial diseases of varying etiology, making it difficult to diagnose the underlying disease. Serological testing for autoimmune diseases may indicate the appropriate direction for further diagnostics. On the other hand, establishing a precise diagnosis of the underlying disease may enable optimal treatment and, thus, potentially affect its course and prognosis.*

Question 4: **Is the opinion of the multidisciplinary team necessary for establishing a diagnosis of fibrotic interstitial lung disease?**

#### 3.1.4. Background

Current international recommendations for IPF diagnosis, as well as the increasing number of published national recommendations, indicate the benefits of a multidisciplinary discussion (MDD) in the diagnostic process of every patient undergoing diagnostics due to an ILD of unknown etiology with signs of fibrosis and clinical suspicion of IPF [5,7,27,45,46,47,48]. The consistency between a single-discipline decision (SDD), i.e., a decision established by one specialist or a group of physicians in one specialty, and an MDD is rated at 70% (47–87%) [49,50,51,52,53]. A multidisciplinary approach may prevent the implementation of inappropriate treatment, delay in appropriate therapy, and unnecessary and potentially dangerous or costly diagnostic procedures [5,54,55]. A multidisciplinary team involved in the diagnosis of fibrotic ILDs should always include a clinician—a pneumonologist and radiologist—as well as a pathologist (in the case of a lung biopsy)—all with sufficient experience in the diagnosis of ILDs. It is estimated that autoimmune origin can play a role in around 25% of ILD patients, and an ILD may be the first or the dominant sign of systemic disease [6,12,13,40,56]. Many authors, therefore, point out a significant improvement in the diagnostic efficacy of such a team if it includes a rheumatologist [57,58,59,60]. If necessary, a specialist in occupational medicine (potential environmental exposure), pathophysiologist, cardiologist (supportive, e.g., in determining the risk of invasive diagnostics and pulmonary hypertension management), thoracic surgeon, or other specialists, whose assessments may be useful in diagnostic or therapeutic decisions, can join the team [5,27,45,46,61]. The role of MDDs, especially with regard to diagnosing fibrotic ILDs, is emphasized when there is no definite radiological diagnosis of UIP (on HRCT) [5,47,62]. Expert panel recommendations have been depicted in Box 4.

Box 4 **Diagnostic Module Recommendation 4.**

**Recommendation 4:**

**We recommend that an opinion of a multidisciplinary team should be taken into account in the diagnostics of fibrotic interstitial lung disease**.

*Evidence quality: very low*

**Strength of recommendation: strong**

(voting results: strongly for—19 votes, conditionally for—7 votes, abstain from voting—0 votes, conditionally against—3 votes, strongly against—0 votes)

**Commentary:**  *The diagnostic process for a fibrotic ILD is multidirectional, multifaceted, and multistage, and its integration requires specialist knowledge in many areas of medicine. A structured, multidimensional attitude toward make such a decision increases the likelihood of establishing a correct diagnosis, potentially shortens the time of this process, which may affect the improvement of the diagnostic process and the possibility of using an optimal treatment, and, thus, potentially influences the long-term prognosis.*

Question 5: **Should a patient with an interstitial lung disease other than IPF who does not meet the criteria for the progressive fibrosing phenotype be monitored for progression?**

#### 3.1.5. Background

For the purposes of the INBUILD trial, an arbitrary criterion of up to 24-month follow-up was adopted, during which a confirmation of ILD progression, despite standard treatment, constituted an inclusion criterion [16]. In order to define the population of progressive ILDs, other studies assumed periods of follow-up ranging between 6 and 24 months [63]. Based on everyday practice, we know that disease progression can occur at any time, including after temporary stabilization. Data from the literature on the natural history of interstitial diseases and the trajectory of radiological and functional changes during longer follow-up periods are very scarce. Observations of patients with SSc-ILD indicate that approximately one-third of patients experience ILD progression during the first 12 months of follow-up, while up to 67% experience it within 5 years on average [64]. The retrospective PROGRESS study provided valuable information on a group of 165 patients with interstitial diseases other than IPF who were followed for 8 years. It was demonstrated that the time between ILD diagnosis and fulfilling criteria for disease progression was 2 years (interquartile range (IQR): 0–3.3 years), with the longest time from diagnosis to progression of 20.8 years. Progression was reported as >10% loss of FVC in 66% of cases [65]. In another retrospective study with a median follow-up of 62.7 months, the authors pointed at risk factors for progression, such as diagnosis of hypersensitivity pneumonitis and lower baseline FVC and TLC values [10]. Monitoring should include an assessment of signs and symptoms, a broad range of lung function tests (e.g., FVC, T_L_,_CO_), and, in justified cases, HRCT. There are no guidelines on the frequency of follow-up investigations for ILDs. It seems reasonable to plan clinical and functional assessments every 6–12 months, with radiological tests (HRCT) performed every 12–24 months. In selected cases, when symptoms worsen or risk factors for progression are present, individual monitoring should be planned. Expert panel recommendations have been depicted in Box 5.

Box 5 **Diagnostic Module Recommendation 5.**

**Recommendation 5:**

**We recommend that a patient with an interstitial lung disease other than IPF who does not meet the criteria for the progressive fibrosing phenotype should be monitored for progression**.

*Evidence quality: very low*

**Strength of recommendation: strong**

(voting results: strongly for—19 votes, conditionally for—10 votes, abstain from voting—0 votes, conditionally against—0 votes, strongly against—0 votes)

**Commentary:**  *The time criteria used in the current literature to define PF-ILDs typically limit the follow-up time to 24 months. However, it is known from clinical observations that progression may occur even after many years of stable disease. It is of note that PF-ILD diagnosis does not always become an indication for anti-fibrotic therapy.*

Question 6: **Should patients with systemic autoimmune diseases be monitored regularly for signs of interstitial lung disease?**

#### 3.1.6. Background

ILDs may occur at any stage in the development of systemic autoimmune disease. The risk of ILDs varies depending on the definition, assessment methods, time of observation, and type of the underlying disease, and it often also depends on the types of autoantibodies present [66]. Diseases with the highest incidence of interstitial lung lesions include systemic sclerosis (35% based on retrospective registry analysis) [67], mixed connective tissue disease (52% of patients in HRCT) [68], and idiopathic inflammatory myopathies (20–86% of patients) [69]. Slightly less frequent ILDs develop in people with rheumatoid arthritis (7.7% in a long-term population-based observational study) [70], Sjögren’s syndrome (20%) [71], or systemic lupus erythematosus (1–15%) [72]. Interstitial changes may resemble non-specific interstitial pneumonia (NSIP), usual interstitial pneumonia (UIP), organizing pneumonia (OP), or lymphocytic interstitial pneumonia (LIP). Only a proportion of cases occur with dominant interstitial fibrosis, which is not progressive in all patients. The autoantibodies associated with the highest risk of development of interstitial lung lesions are: anti-Jo-1, anti-PL7, anti-PL12, anti-SSA/Ro52, anti-MDA5, anti-Scl70, anti-PMScl, and anti-Th/To [73]. Risk factors for ILDs in patients with systemic autoimmune diseases vary depending on the diagnosis, e.g., in rheumatoid arthritis (RA), these include male gender, smoking, older age, anti-CCP antibodies, and longer disease duration; in systemic sclerosis, these include: male gender, autoantibodies against topoisomerase I (anti-Scl70 antibodies), or diffuse sclerosis [73]. 

Due to the high incidence of ILDs in the population of patients with a systemic autoimmune disease, it is recommended that patients be monitored for symptoms suggestive of interstitial lesions [5]. Alarming symptoms include: the appearance of bibasilar crackles audible on auscultation of the lungs, the development of a dry cough, or shortness of breath on exertion [73]. 

ILDs may, in some cases, have a rapid course and be associated with a poor prognosis [72]. In systemic sclerosis, pulmonary fibrosis is a major cause of mortality [74]. An ILD can be one of the first symptoms (e.g., in antisynthetase syndrome) [41] or may occur after many years of the disease (e.g., in RA) [75], significantly reducing patients’ quality of life and worsening prognosis [76]. Active anti-nicotine education can reduce the risk of its development [77], and its early diagnosis and initiation of therapy with documented efficacy are essential for slowing disease progression [77]. Expert panel recommendations have been depicted in Box 6.

Box 6 **Diagnostic Module Recommendation 6.**

**Recommendation 6:**

**We recommend that patients with systemic autoimmune diseases should be regularly monitored for signs of interstitial lung disease**.

*Evidence quality: very low*

**Strength of recommendation: strong**

(voting results: strongly for—19 votes, conditionally for—9 votes, abstain from voting—0 votes, conditionally against—1 vote, strongly against—0 votes)

**Commentary:**  *The significant likelihood of pulmonary manifestations of systemic disease warrants the need for regular monitoring of its occurrence in each case. The scope of investigations performed and the time intervals should be individualized and should take into account the diagnosis, clinical presentation, and risk factors. For instance, in SSc, we propose HRCT and PFT every 12 months or earlier if needed.*

### 3.2. Treatment Module

Question 1: **Should patients with an ILD in the course of systemic autoimmune disease be managed by a multidisciplinary team?**

#### 3.2.1. Background

Given the broad spectrum of ILDs and their differentiation in terms of etiology, pathogenesis, clinical presentation, and long-term prognosis, AI-ILD and interstitial pneumonia with autoimmune features (IPAF) deserve special attention in this aspect. Interstitial patterns in these entities may include NSIP, UIP, OP, or LIP. Immunosuppressants and glucocorticosteroids used in the treatment of systemic autoimmune disease may insufficiently control the course of an ILD. Moreover, anti-fibrotic drugs do not affect extrapulmonary manifestations [23]. On the other hand, as demonstrated in the SENSCIS study, the combination of an anti-fibrotic with a mycophenolate mofetil may provide beneficial effects on slowing the rate of progression of interstitial lung fibrosis [23]. The development of the optimal treatment regimen should be the result of a joint discussion of the representatives of various specialists—in this case, a pneumonologist and rheumatologist with appropriate clinical experience. Consideration should be given to the possibility of combining drugs of different pharmacologic classes; a decision on optimal treatment (if monotherapy is considered) must be made, or treatment should be discontinued if an MDD asserts that the treatment will not bring the expected benefits and potential adverse effects will worsen the quality of life. Making these decisions is particularly difficult given the lack of data on potential interactions between different drug groups [78,79,80]. In the event of such doubts, the composition of the multidisciplinary team should be extended to include a clinical pharmacologist. Agreeing on the optimal treatment regimen, examinations, and follow-up visits is also a task for the multidisciplinary team [4,16,81]. Decisions on non-pharmacological treatment (palliative care, lung transplantation) may require the participation of specialists in the relevant fields. Expert panel recommendations have been depicted in Box 7.

Box 7 **Treatment Module Recommendation 1.**

**Recommendation 1:**

**We recommend that an opinion of a multidisciplinary team should be considered in the management of patients with interstitial lung disease in the course of systemic autoimmune diseases**.

*Evidence quality: very low*

**Strength of recommendation: strong**

(voting results: strongly for—22 votes, conditionally for—5 votes, abstain from voting—0 votes, conditionally against—2 votes, strongly against—0 votes)

**Commentary:**  *Systemic autoimmune diseases are associated with extrapulmonary manifestations, which often represent a comparable or more significant clinical problem than manifestations of respiratory involvement. Therefore, in these cases, an opinion made by an experienced clinician, rheumatologist, and, depending on the needs, other specialists is necessary.*

Question 2: **Should a patient with a progressive fibrosing interstitial lung disease (PF-ILD) other than IPF be treated with first-line therapy dedicated to the diagnosed underlying disease?**

#### 3.2.2. Background

A progressive interstitial pulmonary fibrosis phenotype other than IPF occurs in different disease entities, such as HP, AI-ILD, iNSIP, and uILD [65,82]. Fibrosis in PF-ILD is often preceded by or related to activation of various inflammatory and fibrotic pathways that may lead to fibroblast activation and differentiation into myofibroblasts, producing an extracellular matrix, which results in the remodeling of the lung parenchyma and leads to pulmonary fibrosis [83]. In the treatment of these ILDs, glucocorticosteroids or immunosuppressants (e.g., cyclophosphamide, azathioprine, mycophenolate mofetil, rituximab) are used. The impact of immunosuppression on PF-ILD is largely unknown, except for ILD in systemic sclerosis (SSc-ILD) [84]. Randomized clinical trials of SSc-ILD showed that cyclophosphamide-treated patients achieved a slower decline in FVC after one year of treatment compared to the placebo group, and a study assessing the efficacy of two years of mycophenolate mofetil (MMF) treatment vs. cyclophosphamide treatment showed that the effects of the two drugs were comparable, with lower toxicity of MMF [85,86]. Immunomodulatory treatment of the underlying disease may be of major importance, particularly in autoimmune diseases, which should take into account not only respiratory effects, but also the overall disease activity and inflammatory processes in other organs and tissues [87]. 

In the treatment of HP, the first necessary step is to identify and eliminate the causal antigen, which has a positive impact on the course of the disease and prognosis [4,34]. Early immunomodulatory treatment in patients with fibrosing NSIP or HP may be associated with improved respiratory function and a favorable long-term prognosis [19,20]. MMF and azathioprine are considered first-choice drugs in patients with fibrosing HP who present with disease progression despite previous glucocorticoid therapy [83]. A study evaluating the effect of MMF or azathioprine on lung function in patients with chronic HP demonstrated that both drugs were well tolerated and reduced the need for prednisone, and that annual treatment significantly improved T_L,CO_ [88]. However, it should be noted that other studies showed opposite results [89,90]. Expert panel recommendations have been depicted in Box 8.

Box 8 **Treatment Module Recommendation 2.**

**Recommendation 2:**

**We suggest that a patient with a progressive fibrosing interstitial lung disease (PF-ILD) other than IPF should be treated with first-line therapy dedicated to the diagnosed underlying disease**.

*Evidence quality: very low*

**Strength of recommendation: conditional**

(voting results: strongly for—12 votes, conditionally for—12 votes, abstain from voting—5 votes, conditionally against—0 votes, strongly against—0 votes)

**Commentary:**  *Treatment of the underlying disease—in particular, diseases in which the respiratory system is just one possible manifestation—may have a potentially beneficial effect on the course of interstitial lung disease and improve long-term prognosis. The results of single randomized clinical trials indicate efficacy in limiting AI-ILD progression.*

Question 3: **In a patient with a progressive fibrosing interstitial lung disease (PF-ILD) other than IPF, should anti-fibrotic therapy with nintedanib be used in the event of the ineffectiveness of the therapy recommended for the treatment of the underlying disease?**

#### 3.2.3. Background

Treatment of ILDs other than IPF with a predominant component of inflammation from the point of view of the disease pathobiology is currently based on immunomodulatory therapy (glucocorticoids or immunosuppressants) and elimination of known causal factors in occupational or environmental diseases [4,91]. In practice, decisions on optimal immunomodulatory treatment are driven mainly by the diagnosis of the underlying disease and its course. Nevertheless, a considerable percentage of patients will, despite the use of immunomodulatory therapy recommended for the treatment of the underlying disease, develop PF-ILD, regardless of the initial diagnosis of an ILD. In these cases, from the point of view of pathobiology, the dominant factor is the process of fibrosis, rather than inflammation, and at the same time, this determines the progression of the disease, although the specific mechanisms responsible for the development of this phenotype are not known [92]. In this situation, immunomodulatory treatment is likely ineffective in terms of ILD control and is unable to prevent further worsening of the patient’s clinical condition. A recently completed randomized phase III INBUILD clinical trial demonstrated the efficacy and safety of anti-fibrotic therapy with nintedanib in the population of patients with PF-ILDs other than IPF [16]. The benefits of nintedanib were also demonstrated in both the overall study population and in subgroups of patients with UIP and non-UIP radiological patterns [16]. Even though the INBUILD study did not have the power to provide evidence in favor of nintedanib in specific diseases in a broad spectrum of PF-ILDs, its results suggest that nintedanib reduces the rate of progression of ILDs measured by the decline in FVC in patients with PF-ILDs, regardless of the initial ILD diagnosis [18]. At the same time, additional analyses of the study showed that concomitant use of glucocorticoids at the initiation of nintedanib treatment or the addition of other immunomodulatory therapies during treatment did not adversely affect the benefits of nintedanib in reducing the rate of decline in FVC [81], and the benefits were consistent regardless of the progression criterion used in the identification of the PF-ILD [93] or baseline FVC [94]. Expert panel recommendations have been depicted in Box 9.

Box 9 **Treatment Module Recommendation 3.**

**Recommendation 3:**

**We recommend that in a patient with a progressive fibrosing interstitial lung disease (PF-ILD) other than IPF, anti-fibrotic therapy with nintedanib should be used in the event of ineffectiveness of the therapy recommended for the treatment of the underlying disease**.

*Evidence quality: low*

**Strength of recommendation: strong**

(voting results: strongly for—16 votes, conditionally for—10 votes, abstain from voting—2 votes, conditionally against—1 vote, strongly against—0 votes)

**Commentary:**  *This recommendation promotes the use of nintedanib as the only treatment option currently available.*

Question 4: **In a patient with a progressive fibrosing interstitial lung disease (PF-ILD) other than IPF, should anti-fibrotic therapy with pirfenidone be used in the event of ineffectiveness of the therapy recommended for the treatment of the underlying disease?**

#### 3.2.4. Background

Pirfenidone is the first approved treatment for IPF [95,96,97]. Given its multifactorial anti-fibrotic effect, a similar effect may also be expected in patients with other ILDs with the progressive fibrosis phenotype. We have the results of several randomized clinical trials. The RELIEF study enrolled patients with progressive pulmonary fibrosis in AI-ILD, NSIP, fHP and asbestos exposure [98]. Most patients received standard treatment with glucocorticoids alone or in combination with an immunosuppressant. The study was terminated early due to too slow enrollment of eligible patients. The analysis of available data showed that patients receiving pirfenidone had a significantly lower FVC decline than patients receiving a placebo (difference between the groups of 1.69%, *p* = 0.042). No significant differences in progression-free survival were demonstrated, while a higher proportion of patients on pirfenidone maintained stable functional parameters (FVC decline of less than 5% over 48 weeks). The beneficial effects of pirfenidone were also observed with regard to T_L_,co and distance in 6MWT [98]. The second phase (II), a multicenter, international, randomized, double-blind, placebo-controlled study, investigated the efficacy of pirfenidone in patients with unclassifiable ILDs with the progressive fibrosing phenotype [17]. The primary endpoint was a change in FVC after 24 weeks of treatment as assessed by daily home spirometry. It was not achieved due to technical difficulties, irregularities, and lack of consistency in performing this examination by patients at home. However, the evaluation of office spirometry (secondary endpoint) showed a smaller decline in FVC in pirfenidone-treated patients as compared with the placebo (17.8 mL vs. 113.0 mL/24 weeks). Fewer patients in the pirfenidone group experienced FVC declines greater than 5 and 10% over the study duration. No differences in progression-free survival or quality of life were observed. Although the aforementioned studies did not meet formal requirements, they indicate the efficacy of pirfenidone in inhibiting pulmonary fibrosis progression, as in the case of IPF. No new safety signals were observed—the adverse event profile was consistent with the one observed in the studies in IPF patients [17,98]. Pirfenidone is currently being studied in patients with SSc-ILD, rheumatoid-arthritis-associated interstitial lung disease (TRAIL-1), sarcoidosis with pulmonary fibrosis, fHP, and pneumosilicosis [99]. Expert panel recommendations have been depicted in Box 10.

Box 10 **Treatment Module Recommendation 4.**

**Recommendation 4:**

**No recommendations were made for or against the use of anti-fibrotic therapy with pirfenidone if treatment of the underlying disease has failed in a patient with a progressive fibrosing interstitial lung disease (PF-ILD) other than IPF**.

*Evidence quality: very low*

**Strength of recommendation: not issued**

(voting results: strongly for—1 vote, conditionally for—12 votes, abstain from voting—14 votes, conditionally against—2 votes, strongly against—0 votes)

**Commentary:**  *It should be noted that the lack of a recommendation does not mean a negative recommendation. Some patients with PF-ILDs other than IPF may benefit from pirfenidone treatment.*

Question 5: **Is it possible to use an anti-fibrotic agent as a first-choice therapy (without the need for previous immunomodulatory treatment) in certain clinical situations (UIP or fibrotic NSIP pattern)?**

#### 3.2.5. Background

Currently, no studies are available that directly assess the efficacy of immunomodulatory treatment compared to anti-fibrotic therapy in patients with PF-ILDs. Treatment decisions in this group of patients are, therefore, difficult and should be supported by a discussion in a multidisciplinary team, considering close collaboration with a rheumatologist in the case of AI-ILD. Antifibrotic therapy as a first-choice therapy should be considered in patients with an IPF-like phenotype, i.e., patients with a UIP pattern in lung HRCT or histopathological examinations and presenting worsening respiratory symptoms, FVC decline ≥ 10% within 12 months, and especially in those patients for whom immunosuppressive therapy would be associated with greater potential adverse effects [83,100]. The presence of the UIP pattern in patients with RA-associated ILD or fibrotic HP is associated with a worse prognosis than in the case of other patterns visible in HRCT and histology [100,101]. A comparison of the placebo groups from the INPULSIS and INBUILD studies showed that the FVC decline was similar between IPF and PF-ILD patients with a similar pattern of UIP in HRCT [3]. Immunosuppressive therapy in patients with IPF was associated with poorer survival compared with the placebo [102,103]. Some retrospective studies also suggested the deleterious effects of immunosuppressive therapy in fHP [89,90].

Patients with fibrosing NSIP have a poorer prognosis than patients with the cellular disease [104]. Immunomodulatory treatment (glucocorticoids, MMF, azathioprine, cyclophosphamide, rituximab) is the treatment of choice in NSIP patients according to the previous recommendations [104,105]. Antifibrotic agents were evaluated in randomized clinical trials in patients with PF-ILD and SSc-ILD, some of which included patients with fibrotic NSIP [16,23,37]. Studies with nintedanib have shown that it slowed the rate of decline in FVC by 57% in PF-ILD, with 19% being patients with NSIP, and by 44% in SSc-ILD, where NSIP was the predominant form of ILD [16,23]. 

Treatment with nintedanib, the only agent currently approved for the treatment of PF-ILD, should be considered in the case of fibrosis progression in patients with NSIP when immunosuppressive therapy is contraindicated. Expert panel recommendations have been depicted in Box 11.

Box 11 **Treatment Module Recommendation 5.**

**Recommendation 5:**

**We suggest using an anti-fibrotic agent as the first-choice treatment in certain clinical situations**.

*Evidence quality: very low*

**Strength of recommendation: conditional**

(voting results: strongly for—7 votes, conditionally for—12 votes, abstain from voting—6 votes, conditionally against—4 votes, strongly against—0 votes)

**Commentary:**  *Immunosuppressive therapy is ineffective and even harmful in patients with IPF. Some patients with interstitial fibrosis with a UIP pattern are unlikely to have a positive response to such treatment. In patients in whom fibrotic lung lesions predominate in the presentation or are the only manifestation of the disease, it is reasonable to initiate anti-fibrotic treatment without immunomodulatory treatment.*

Question 6: **Can a patient with a progressive fibrosing interstitial lung disease (PF-ILD) other than IPF receive simultaneous treatment with a disease-modifying drug and anti-fibrotic therapy?**

#### 3.2.6. Background

Immunosuppressive therapy remains the basis for the management of patients with PF-ILDs—in particular, autoimmune diseases, such as rheumatoid arthritis or systemic sclerosis. Glucocorticosteroids and certain immunosuppressive agents, such as MMF and azathioprine, are also used in the treatment of HP, NSIP, or uILD [14,106]. Despite such treatments, approximately 18 to 32% of patients with ILD other than IPF are estimated to develop a progressive fibrosing phenotype [14]. The results of randomized clinical trials in recent years show a beneficial effect of anti-fibrotic drugs on slowing the rate of progression of PF-ILDs [16,23,98]. Combining immunosuppressive and anti-fibrotic therapy may be a beneficial therapeutic option, taking into account the potential for both therapies to influence different pathogenic pathways involved in the development and progression of PF-ILDs. The safety of a combination treatment with pirfenidone and MMF, as well as nintedanib and MMF, was established in clinical trials in patients with uILD and SSc-ILD [17,23]. The SENSCIS study enrolled patients with SSc-ILD taking MMF at a stable dose in the previous 6 months, methotrexate, or ≤10 mg prednisone, and ultimately, around half of the patients were treated with MMF. It was observed that in the placebo group, the decline in FVC was lower in patients receiving MMF, suggesting a potentially beneficial effect of MMF. In addition, patients treated with both nintedanib and MMF had the slowest rate of FVC decline, suggesting a potential role for combination therapy in SSc-ILD [23]. 

In the INBUILD trial, immunosuppressant use was not allowed at randomization and for the next 6 months, except for prednisone at a dose of ≤20 mg/day. Post-study analysis showed that the use of glucocorticoids at baseline or the initiation of immunomodulatory treatment during the study had no impact on the beneficial effects of nintedanib in patients with PF-ILDs [81]. An SLS-III trial evaluating the efficacy and safety of the combination therapy with pirfenidone and MMF versus MMF alone in patients with SSc-ILD is ongoing [99]. Expert panel recommendations have been depicted in Box 12.

Box 12 **Treatment Module Recommendation 6.**

**Recommendation 6:**

**We suggest that in a patient with a progressive fibrosing interstitial lung disease (PF-ILD) other than IPF, one should consider simultaneous treatment with a disease-modifying drug and anti-fibrotic therapy**.

*Evidence quality: very low*

**Strength of recommendation: conditional**

(voting results: strongly for—6 vote, conditionally for—17 votes, abstain from voting—5 votes, conditionally against—1 vote, strongly against—0 votes)

**Commentary:**  *Taking into account the potential benefits of adding an anti-fibrotic agent to background therapy, such management should be considered in patients with PF-ILDs, particularly in patients with systemic autoimmune disease.*

Question 7: **Should progression noted during treatment with an anti-fibrotic agent in a patient with a progressive fibrosing interstitial lung disease (PF-ILD) other than IPF be a reason for discontinuation of anti-fibrotic therapy?**

#### 3.2.7. Background

The results of the INBUILD study showed that anti-fibrotic therapy with nintedanib slowed the rate of FVC loss in a population of patients who developed progressive fibrosis (PF-ILD) in interstitial diseases other than IPF (non-IPF ILD) [16]. Moreover, an additional post hoc analysis of the study’s results in the overall patient population that evaluated the predicted categorical absolute changes in FVC percentage (FVC%) over a 52-week study period showed that the percentage of patients experiencing clinically meaningful declines in FVC% (FVC decline ≥5%) was lower in the nintedanib group compared with that in the placebo group [107,108]. Currently, there are no data on the efficacy of nintedanib in patients with PF-ILDs beyond 52 weeks or data indicating the benefit of continuing anti-fibrotic treatment in patients with PF-ILDs who experience disease progression during such treatments. Data on the prognostic significance of FVC decline in the non-IPF ILD population are scarce. At the same time, studies in the IPF population have shown that FVC decline is a weak predictor of future FVC decline despite its association with mortality [109,110,111]. Published analyses of pooled data from registration trials for anti-fibrotic agents in IPF provided evidence that continued pirfenidone therapy benefited patients with IPF who had significant on-treatment disease progression (defined as a decline in FVC of ≥10% over 6 months of treatment), with a risk reduction with respect to further FVC decline or death [110]. A similar analysis of pooled data from the INPULSIS I and II studies suggested the benefit of continued treatment with nintedanib in patients with IPF despite disease progression [111]. Expert panel recommendations have been depicted in Box 13.

Box 13 **Treatment Module Recommendation 7.**

**Recommendation 7:**

**No recommendations were made for or against termination of anti-fibrotic therapy in the case of noted progression during treatment of a progressive fibrosing interstitial lung disease (PF-ILD) other than IPF**.

*Evidence quality: very low*

**Strength of recommendation: not issued**

(voting results: strongly for—4 votes, conditionally for—6 votes, abstain from voting—9 votes, conditionally against—8 votes, strongly against—2 votes)

**Commentary:**  *In each case of PF-ILD progression, the decision should be made on an individual basis.*

Question 8: **Should the same principles of non-pharmacological and palliative treatment and eligibility for lung transplantation be applied in a patient with an interstitial lung disease other than IPF with progressive fibrosis as in a patient with IPF?**

#### 3.2.8. Background

Non-pharmacological treatment of pulmonary diseases is a significant addition to pharmacological treatment regardless of diagnosis.

Pulmonary rehabilitation is an important component of the support for the maintenance of function and quality of life of patients with IPF [112,113,114,115]. The number of randomized rehabilitation trials was low, and they included patients not only with IPF, but also with other ILDs, including those associated with systemic autoimmune diseases, NSIP, sarcoidosis, and HP [116,117,118,119,120,121]. Patients with ILDs other than IPF benefit from rehabilitation irrespective of the degree of lung function impairment [122,123]. The improvement related to pulmonary rehabilitation was mainly associated with reduced dyspnea, prolonged walking distance, and improved quality of life [124].

Long-term home oxygen therapy is an established supportive therapy for respiratory failure in chronic obstructive pulmonary disease (COPD), and by analogy, it is also used in patients with respiratory failure due to other causes, e.g., ILDs. Exertional hypoxemia is commonly observed in ILD patients, and it necessitates the use of portable oxygen concentrators during exercise. There is no conclusive evidence for the efficacy of such treatment in improving exercise tolerance or survival. Two randomized studies confirmed a positive impact on the quality of life of patients with various ILDs who used portable oxygen concentrators during exercise, but these were studies in small groups of patients [125,126].

Palliative care is focused on reducing the symptoms of the disease and improving the quality of life of patients with chronic lung diseases. Given the symptoms that are common for IPF and other PF-ILDs, such as shortness of breath, cough, reduced exercise tolerance, and pain in some systemic autoimmune diseases, the indications and palliative therapy methods in these patients should not differ from those for IPF patients [127,128,129].

Lung transplantation is a treatment intended for patients with end-stage respiratory diseases with an expected graft-free survival of less than 2 years. The criteria for inclusion on the waiting list for patients with PF-ILDs do not differ from those used in IPF patients. Disease progression despite treatment and severe lung function impairment are the main indications [130]. Expert panel recommendations have been depicted in Box 14.

Box 14 **Treatment Module Recommendation 8.**

**Recommendation 8:**

**We recommend that the same principles of non-pharmacological and palliative treatment and eligibility for lung transplantation should be applied in a patient with an interstitial lung disease other than IPF with progressive fibrosis as in a patient with IPF**.

Evidence quality: very low

**Strength of recommendation: strong**

(voting results: strongly for—16 votes, conditionally for—10 votes, abstain from voting—3 votes, conditionally against—0 votes, strongly against—0 votes)

**Commentary:**  *Taking into account the similarity of the natural history of PF-ILDs to that of IPF and the lack of alternative management options, the application of the same principles of non-pharmacological, palliative, and terminal care seems to be justified.*

## 4. Conclusions

Summary of recommendations in Table 1.

## Figures and Tables

**Table 1:** Summary of recommendations.

Module	Pico Question		Recommendation	Quality of Evidence	Strength of Recommendation
**DIAGNOSIS**	1	Can a combination of data indicating an increased severity of clinical symptoms, worsening of respiratory function parameters, and progression of lesions in high-resolution computed tomography (HRCT) serve as a basis for the diagnosis of progressive fibrosing interstitial lung disease (PF-ILD)?	We suggest that the basis for the diagnosis of progressive fibrosing interstitial lung disease (PF-ILD) should be an increased severity of clinical symptoms, worsening of pulmonary function parameters, and progression of lesions in high-resolution computed tomography of the chest.	Very low	Conditional
	2	In each patient with fibrotic interstitial lung disease, should we aim at establishing a precise diagnosis of the underlying disease?	We recommend that in every patient with fibrotic interstitial lung disease, we should aim for establishing a precise diagnosis of the underlying disease.	Very low	Strong
	3	Is it necessary to perform serological tests for autoimmune diseases in every patient with fibrotic interstitial lung disease of unknown etiology?	We recommend that every patient with fibrotic interstitial lung disease of unknown etiology should undergo serological tests for autoimmune diseases.	Very low	Strong
	4	Is an opinion of a multidisciplinary team necessary for establishing a diagnosis of a fibrotic interstitial lung disease?	We recommend that an opinion of a multidisciplinary team should be taken into account in the diagnostics of fibrotic interstitial lung disease.	Very low	Strong
	5	Should a patient with an interstitial lung disease other than IPF who does not meet the criteria for the progressive fibrosis phenotype be monitored for progression?	We recommend that a patient with an interstitial lung disease other than IPF who does not meet the criteria for the progressive fibrosis phenotype should be monitored for progression.	Very low	Strong
	6	Should patients with systemic autoimmune diseases be monitored regularly for signs of interstitial lung disease?	We recommend that patients with systemic autoimmune diseases should be regularly monitored for signs of interstitial lung disease.	Very low	Strong
**TREATMENT**	1	Should patients with an ILD in the course of systemic autoimmune diseases be managed by a multidisciplinary team?	We recommend that an opinion of a multidisciplinary team should be considered in the management of patients with interstitial lung disease in the course of systemic autoimmune diseases.	Very low	Strong
	2	Should a patient with a progressive fibrosing interstitial lung disease (PF-ILD) other than IPF be treated with first-line therapy dedicated to the diagnosed underlying disease?	We suggest that a patient with a progressive fibrosing interstitial lung disease (PF-ILD) other than IPF should be treated with first-line therapy dedicated to the diagnosed underlying disease.	Very low	Conditional
	3	In a patient with a progressive fibrosing interstitial lung disease (PF-ILD) other than IPF, should anti-fibrotic therapy with nintedanib be used in the event of ineffectiveness of the therapy recommended for the treatment of the underlying disease?	We recommend that in a patient with a progressive fibrosing interstitial lung disease (PF-ILD) other than IPF, anti-fibrotic therapy with nintedanib should be used in the event of ineffectiveness of the therapy recommended for the treatment of the underlying disease.	Low	Strong
	4	In a patient with a progressive fibrosing interstitial lung disease (PF-ILD) other than IPF, should anti-fibrotic therapy with pirfenidone be used in the event of ineffectiveness of the therapy recommended for the treatment of the underlying disease?	No recommendations were made for or against the use of anti-fibrotic therapy with pirfenidone if treatment of the underlying disease has failed in a patient with a progressive fibrosing interstitial lung disease (PF-ILD) other than IPF.	Very low	Not issued
	5	Is it possible to use an anti-fibrotic agent as a first-choice therapy (without the need for previous immunomodulatory treatment) in certain clinical situations (UIP or fibrotic NSIP pattern)?	We suggest using an anti-fibrotic agent as the first-choice treatment in certain clinical situations.	Very low	Conditional
	6	Can a patient with a progressive fibrosing interstitial lung disease (PF-ILD) other than IPF receive simultaneous treatment with a disease-modifying drug and anti-fibrotic therapy?	We suggest that in a patient with a progressive fibrosing interstitial lung disease (PF-ILD) other than IPF, one should consider simultaneous treatment with a disease-modifying drug and anti-fibrotic therapy.	Very low	Conditional
	7	Should progression noted during treatment with an anti-fibrotic agent in a patient with a progressive fibrosing interstitial lung disease (PF-ILD) other than IPF be a reason for discontinuation of anti-fibrotic therapy?	No recommendations were made for or against the termination of anti-fibrotic therapy in the case of noted progression during treatment of a progressive fibrosing interstitial lung disease (PF-ILD) other than IPF.	Very low	Not issued
	8	Should the same principles of non-pharmacological and palliative treatment and eligibility for lung transplantation be applied in a patient with an interstitial lung disease other than IPF with progressive fibrosis as in a patient with IPF?	We recommend that the same principles of non-pharmacological and palliative treatment and eligibility for lung transplantation should be applied in a patient with an interstitial lung disease other than IPF with progressive fibrosis as in a patient with IPF.	Very low	Strong

## Data Availability

Not applicable.
